# Application of Highly Purified Electrolyzed Chlorine Dioxide for Tilapia Fillet Disinfection

**DOI:** 10.1155/2014/619038

**Published:** 2014-02-17

**Authors:** Chen-Hsing Yu, Tzou-Chi Huang, Chao-Chin Chung, Hao-Hsun Huang, Ho-Hsien Chen

**Affiliations:** ^1^Department of Food Science, National Pingtung University of Science and Technology, 1 Shuefu Road, Neipu, Pingtung 91201, Taiwan; ^2^Department of Biological Science and Technology, National Pingtung University of Science and Technology, 1 Shuefu Road, Neipu, Pingtung 91201, Taiwan

## Abstract

This research aimed to develop an electrolysis method to generate high-concentration chlorine dioxide (ClO_2_) for tilapia fillet disinfection. The designed generator produced up to 3500 ppm of ClO_2_ at up to 99% purity. Tilapia fillets were soaked in a 400 ppm ClO_2_ solution for 5, 10, and 25 min. Results show that total plate counts of tilapia, respectively, decreased by 5.72 to 3.23, 2.10, and 1.09 log CFU/g. In addition, a 200 ppm ClO_2_ solution eliminated coliform bacteria and *Escherichia coli* in 5 min with shaking treatment. Furthermore, ClO_2_ and trihalomethanes (THMs) residuals on tilapia fillets were analyzed by GC/MS and were nondetectable (GC-MS detection limit was 0.12 ppb). The results conform to Taiwan's environmental protection regulations and act governing food sanitation.

## 1. Introduction

Chlorine dioxide (ClO_2_) is a strong oxidant widely applied for sterilization, disinfection, and waste-water treatment. It is commonly used on drinking water and environmental disinfection. It was also recommended as a commercial sanitizer to replace electrolyzed oxidizing water [1, 2], chlorine (Cl_2_), hypochlorous acid (HOCl), and hypochlorite (OCl^−^) [3–5]. Contact of chlorine dioxide with organic substances in food or water results in microbial resistance and inactivation, but it also produces four trihalomethane (THM) byproducts, that is, chloroform, bromodichloromethane, dibromochloromethane, and bromoform, which are associated with toxicity and carcinogenesis [6–9]. In Taiwan, tilapia fillets are an important economic product, and it is common practice to use sodium hypochlorite (NaClO) as a disinfecting agent for processing tilapia fillets; however, treatment of this type could lead to serious problems involving residual THMs in treated seafood [4, 10–12]. As for its application for vegetable and fruit disinfection, ClO_2_ gas has been successfully used to disinfect strawberries, lettuce, cabbage, and cucumbers with continuous methods [4, 13–17]. In this work, the bactericidal efficacy of ClO_2_ was evaluated for cleaning tilapia fillets with different cleaning methods.

Commercial ClO_2_ is commonly generated using chemical methods that react sodium chloride, sodium hypochlorite, or sodium chlorate with sulfuric acid or hydrochloride acid [18, 19]. The chemical method of producing ClO_2_ needs a strong acid (pH 2~3), inhibits Cl_2_ hydrolysis, and takes a long time for activation. The yield of ClO_2_ depends on the purity of the raw materials, the catalyst, pH, reaction time, and temperature [20–24]. Furthermore, it was discovered that electrolyzing sodium chlorite can produce highly purified ClO_2_ [25, 26]. Therefore, the objective of this study is to develop novel electrolysis equipment to produce highly purified, low-cost ClO_2_ to disinfect with water, while simultaneously monitoring trihalomethane (THM) residuals on tilapia fillets.

## 2. Materials and Methods

### 2.1. Materials

Tilapia fillets were bought from a local traditional market in Pingtung, Taiwan. The microbiological media used in this study were peptone and tryptic soy agar (TSA) purchased from Difco Laboratories (Detroit, MI, USA); these were prepared according to the manufacturer's specifications. 3 M Coliform and *E. coli* Petrifilm no. 6414 were purchased from Microbiology Products 3 M Health Care (St. Paul, MN, USA).

### 2.2. ClO_**2**_ Electrolysis Equipment (ClO_**2**_ Generator)

**Figure 1:**
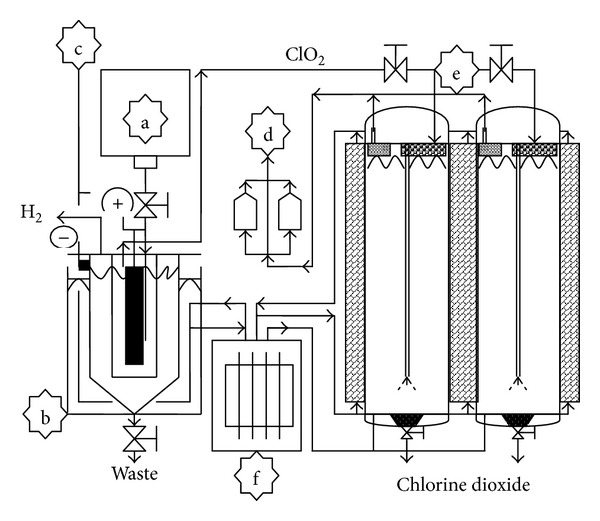
Designed chlorine dioxide electrolysis equipment: (a) material, (b) electrolyzer, (c) electronic control system, (d) air pump, (e) collecting tank, and (f) cooling system.

**Figure 2:**
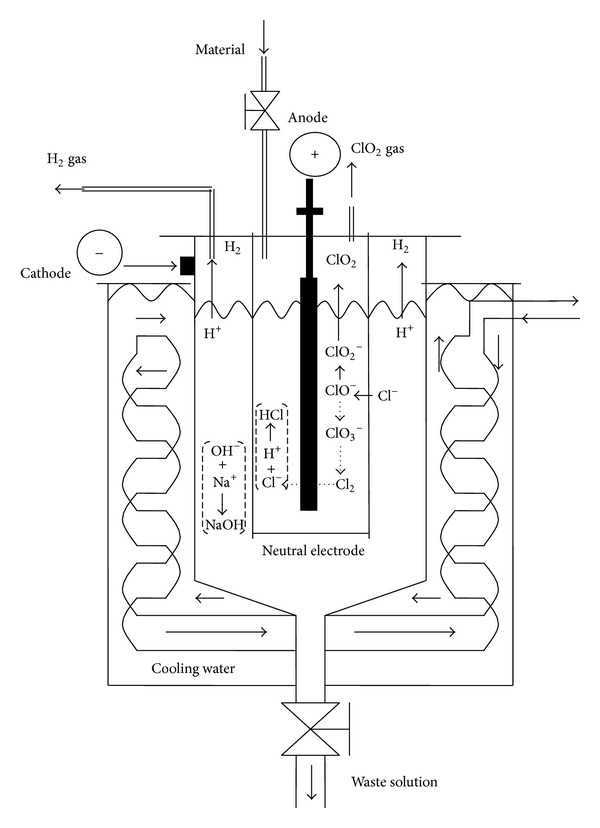
Internal structure and reaction of the electrolysis system.

The self-designed electrolysis equipment consisted of a raw material tank, an electrolyzer, an air pump, two ClO_2_ collecting tanks, and a cooling system (*Taiwan Patent*, no. 200722557) [27].  Figure 1 shows the designed chlorine dioxide electrolysis equipment. The internal structure and reaction of the electrolyzer are shown in Figure 2. Saturated saline and sodium hypochlorite enter and mix in the electrolyzer system using a direct current (100~110 A, 7~8 V), the electrolyzed temperature was controlled to 55~65°C, and the electrolyzed material supply rate was 10 L/h. The temperature of the ClO_2_ collecting tank was maintained at 5~10°C by cooling water from the cooling system. NaCl was electrolyzed into NaClO_2_. The reaction equation is as follows:

(1) $$\begin{array}{c} \text{NaCl}+2{\text{H}}_{2}\text{O}⟶{\text{NaClO}}_{2}+2{\text{H}}_{2}↑. \end{array}$$

Meanwhile, the NaClO_2_ was further electrolyzed, the ClO_2_^−^ was attracted by the cathode, and H_2_O was attracted by the anode to release H_2_ (Figure 2). The reaction equations are as follows:

(2) $$\begin{array}{c} \text{anode}:\;{{\text{ClO}}_{2}}^{-}⟶{\text{ClO}}_{2}↑+{\text{e}}^{-}; \end{array}$$

(3) $$\begin{array}{c} \text{cathode}:\;{\text{H}}_{2}\text{O}+{\text{e}}^{-}⟶{\text{OH}}^{-}+\frac{1}{2}{\text{H}}_{2}; \end{array}$$

(4) overall: 2NaClO2+2H2O         ⟶2ClO2+2NaOH+H2↑

(5) NaCl+NaClO2+4H2O ⟶2ClO2+2NaOH+3H2↑.

The resultant ClO_2_ was aspirated out and collected into 5~10°C pure water in the two collecting tanks. The NaOH solution was collected separately. The oxidation/reduction potential (ORP) and pH of the ClO_2_ solutions were measured using an ORP/pH meter (Mettler-Toledo Seven Easy ORP/pH meter, Kaohsiung, Taiwan).

ClO_2_ analysis: the concentration of ClO_2_ was analyzed using the iodine method [28]. The ClO_2_ solution at 10 mL was diluted 200 times with pure water. It was adjusted to five pH levels and then titrated with a 0.01 N sodium thiosulfite solution. The titration volumes were A, B, C, D, and E. The following calculation formulas were used to calculate the concentrations of ClO_2_, Cl_2_, ClO_2_^−^, and ClO_3_^−^:

(6) ClO2(ppm)=(54)×(B−D)×N ×13,490/sample  volume,

(7) $$\begin{array}{c} {\text{Cl}}_{2}\left(\text{ppm}\right)=\text{D}\times \text{N}\times 16,863/\text{sample}\;\text{volume}, \end{array}$$

(8) ClO2−(ppm)=[E−(A+B)]×N ×13,909/sample  volume,

(9) ClO3−(ppm)=[A−(B−D)4]×N   ×13,909/sample  volume.

Here, N is the concentration of the sodium thiosulfite solution.

### 2.3. Cleaning Methods

Tilapia fillets were incubated at 37°C until the total plate count reached 5~6 log CFU/g. The different purities (45%~99%) of 400 mg/L ClO_2_ solutions were used to test the effect of tilapia disinfection. Tilapia fillets were inoculated with coliforms or *E. coli *at a concentration of 5~6 log CFU/g. At 99% purity, ClO_2_ solutions of 50, 100, and 200 ppm were used to wash the fillets by soaking or shaking treatment for 5, 15, and 25 min, and the total plate counts, coliforms, and *E. coli* of the fillets were determined.

### 2.4. Microbiological Analyses

The total plate count assay followed the China National Standard (CNS 10890 N6186) [29] method: 1 mL of masticated tissue liquid was serially (1 : 10) diluted in 9 mL of 0.1% sterile peptone water, and 0.1 mL portions of appropriate diluents were surface-plated on TSA. The plates were then incubated at 37°C for 48 h in duplicate. CFUs were counted and expressed per gram of sample after logarithmic conversion. The coliform and *E. coli* assays followed Sasithorn and Sirirat [30] using 1 mL of masticated tissue liquid plated on 3 M Petrifilm no. 6414 (St. Paul, MN), and the plates were incubated at 37°C for 24 h in duplicate.

### 2.5. Residual THMs Analyses

The analytical procedure was modified from Stack et al.'s [31] gas chromatographic (GC) method on an Agilent 5890 system coupled to an Agilent 5973N mass spectrometer (MS) (Palo Alto, CA). Chromatographic separation was performed using a capillary column (HP-5, 30 m × 0.32 mm, 0.25 *μ*m phase film thickness) from Agilent Technologies. The initial temperature was 45°C for 3 min and then increased by 8°C/min to a final temperature of 220°C for 20.5 min. The injector temperature was set to 200°C. Nitrogen was used as the carrier gas at a flow rate of 38.5 mL/min.

MS was operated in the electron ionization mode at 70 eV. The mass range was scanned at 40~350 m/z and for 0.60 seconds per scan for the full-scan mode. Temperatures for the trap, manifold, and transfer line were set to 250, 50, and 280°C, respectively. All data for quantification were collected in the selected ion monitoring mode at 83 and 85 m/z for chloroform, 127 and 129 m/z for dibromochloromethane, and 173 m/z for bromoform.

### 2.6. Statistical Analysis

Three replicates were conducted, and each sample was assayed in duplicate. Data collected from the experiments were analyzed by an analysis of variance (ANOVA) and Duncan's multiple range test using the SAS 8.2 program [32]. Significant differences between tested parameters were determined based on a 95% confidence level (*P* < 0.05).

## 3. Results and Discussion

### 3.1. Effects of Different Ratios of NaClO_**2**_ for High Concentrations of ClO_**2**_

Table 1 shows that 10% NaClO_2_ and 20% NaCl generated 4749 ppm of total chlorine and 99.8% pure ClO_2_, respectively. The pH was 2.18, and ORP was 1440 mV (Table 1). When the purity of ClO_2_ varied from 99.5% to 99.8%, the pH increased from 2.36 to 2.18. Under an acidic condition, Cl_2_ easily disassociated into Cl^−^, and ClO_2_ mainly disassociated into ClO_2_^−^ and ClO_3_^−^, with a small portion disassociating into Cl^−^ [21, 33, 34]. A great quantity of Cl^−^ resulted from excessive NaCl in the raw materials which contained NaCl and sodium hypochlorite. At the anode side, NaCl was converted into NaClO_2_ and then into NaClO_3_. NaClO_3_ was affected by the reducing reaction from the cathode side, producing Cl_2_, Cl^−^ and H^+^. The Cl^−^, and H^+^ then formed into very small amounts of HCl. These reaction cycles generated NaOH and ClO_2_, producing Cl_2_.

### 3.2. Effect of ClO_**2**_ Purity on Tilapia Fillet Disinfection

Tilapia fillets were soaked in 400 ppm of 99% pure ClO_2_ for 5, 15, and 25 min. Results indicated that total plate counts on tilapia fillets decreased from 5.72 log CFU/g to 3.23, 2.1, and 1.09 log CFU/g, respectively (Table 2). Although ClO_2_ solutions contained 45%, 50%, and 60% of freely available chlorine, the bactericidal effect was not so obviously effective. One of the explanations could be that the Cl_2_ is not as effective as ClO_2_, because active oxygen molecules diminish the number of electrons on biological cell membranes and cause damage to biological enzymes on biological membranes therefore amino acid and nucleic bodies are hindered from generating proteins in biological cells [33, 35, 36]. Another reason could be that ClO_2_ not only reacts with electrons on biological cell membranes but also reacts with Cl_2_ to achieve disassociation and oxidation under an acidic condition and then forms ClO_2_^−^,  ClO_3_^−^, and Cl^−^ byproducts [21, 33]. The less Cl_2_ there was, the higher the disinfection effect was.

### 3.3. Effect of Various Treatments

Three different concentrations (50, 100, and 200 ppm) of 99% ClO_2_ solutions were used for soaking or shaking disinfection treatment on tilapia fillets for 5, 15, and 25 min. Results are shown in Table 3. After the fish fillets were shaken in the solutions for 5 min, total plate counts were 3.49, 2.41, and 1.29 log CFU/g, respectively, and all were nondetectable after 15 and 25 min, compared to the control groups at 5.84~5.78 log CFU/g (control).

Similar results for coliforms and *E. coli* are shown in Tables 4 and 5. The control groups of coliform (control) were 5.23, 5.29, and 5.25 CFU/g. When tilapia fillets were treated with 50, 100, and 200 ppm of high-purity ClO_2_ solutions with the shaking method for 5 min, the coliform counts, respectively, decreased to 1.78, 1.07 log CFU/g, and nondetectable (Table 4). *Escherichia coli* also showed a > 4 log reduction after 5 min and was nondetectable after 15 and 25 min of shaking (Table 5). Both the soaking and shaking methods eliminated microbial populations; however, the results show that the soaking method was not as effective as the shaking method. Microorganisms attached to fish skin may more easily be washed out by shaking with mechanical forces [37]. Aloisio and Francisco [38] claimed that ClO_2_ being bound to water molecules by static attraction forces under a steady state hindered the bactericidal effect.

### 3.4. Detection of THMs

THM residuals are a problem for the safety of chlorine-treated food materials [39, 40]. After tilapia fillets were washed by soaking or shaking in the ClO_2_ solution with the highest concentration (200 ppm) for 25 min, the waste solutions were analyzed for THMs using GC/MS. THMs include chloroform, dichloromethane, and methyl chloride. The results show that no THMs were detected in a used ClO_2_ solution after soaking (GC-MS detection limit was 0.12 ppb), as shown in Table 6. These results conform to Taiwan's environmental protection regulations and act governing food sanitation. Furthermore, a LC-MS analysis also showed that when using a 200 ppm bactericide solution for 25 min at 25°C, residual of ClO_2_ solution was no detected in solution (the instrument detection limit was 0.1 ppb).

## 4. Conclusions

The results demonstrated the feasibility of stably producing ClO_2_ using electrochemical technology. The maximum concentration and purity of ClO_2_ were obtained when using a mixture that blended 20% NaCl and 7%~10% NaClO_2_ together as the electrolytes. The concentration and purity of ClO_2_ were 3200~4700 ppm and 99.5~99.7%, respectively. Disinfection results indicate that a 200 ppm ClO_2_ solution reduced the total bacterial, coliform, and *E. coli* counts on tilapia fillets by 3.0~4.0 log CFU/g (*P* < 0.05). The soaking wash treatment was more effective than the shaking method. A GC-MS analysis also showed that when using a 200 ppm bactericide solution for 25 min, residual THMs of the ClO_2_ solution were nondetectable. Bactericidal treatment with a ClO_2_ solution for tilapia fillets also conforms to Taiwan's environmental protection regulations and act governing food sanitation. The ClO_2_ solution is indeed a safer method for treating seafood, and our novel electrolysis equipment can produce highly purified, low-cost ClO_2_ to disinfect with water, for immediate use for agricultural product and seafood treatment.

## Figures and Tables

**Table 1:** Constituents of chlorine dioxide aqueous solution on different constituent ratios of electrolytes.

Electrolyte constituent ratio	20% NaCl			
	7% NaClO_2_	8% NaClO_2_	9% NaClO_2_	10% NaClO_2_
Constituents of chlorine dioxide aqueous solution				
ClO_2 _(ppm)	3193.76^d^	3612.31^c^	4232.49^b^	4738.36^a^
ClO_2_^−^ (ppm)	16.02^a^	ND	ND	ND
ClO_3_^−^ (ppm)	ND	ND	ND	ND
Cl_2_ (ppm)	ND	9.75^b^	10.64^a^	10.64^a^
Total chlorine* (ppm)	3209.78^d^	3622.06^c^	4243.12^b^	4749.00^a^
ClO_2_ purity** (%)	99.50^a^	99.73^a^	99.75^a^	99.78^a^
pH (unit)	2.36^a^	2.34^a^	2.38^a^	2.18^b^
Oxidation reduction potential (millivolt)	1.32^c^	1.38^b^	1.44^a^	1.44^a^

^a–d^Means in the same column followed by different superscripts are significantly different at *P* < 0.05 (Duncan's multiple range test).

*Total chlorine (ppm) = ClO_2_ + ClO_2_^−^ + ClO_3_^−^ + Cl_2_.

**ClO_2_ purity = [ClO_2_/(ClO_2_ + ClO_2_^−^ + ClO_3_^−^ + Cl_2_)] × 100%.

**Table 2:** Total bacterial counts of tilapia fillets for various purities in 400 ppm chlorine dioxide solutions.

Treatment time (min)	ClO_2_ purity (%)						
	45%	50%	60%	70%	80%	90%	99%
	Total bacteria count log (CFU/g)*						
5	5.60^a^	5.51^a^	4.53^b^	4.38^c^	4.13^d^	3.96^e^	3.23^f^
15	5.44^a^	5.43^a^	4.3^b^	4.09^c^	3.98^d^	3.29^e^	2.1^f^
25	5.33^a^	4.3^b^	3.13^c^	2.91^b^	2.74^e^	2.54^f^	1.09^g^

^a–g^Means in the same column followed by different superscripts are significantly different at *P* < 0.05 (Duncan's multiple range test).

*Original microbial load: 5.72 log (CFU/g).

**Table 3:** Total bacterial counts for tilapia fillets disinfected by soaking or shaking treatments at different chlorine dioxide concentrations.

Washing method	Concentration (ppm)	Cleaning time (min)		
		5	15	25
		Total bacteria count log (CFU/g)		
Soaking	Control	5.84^ax^	5.80^ax^	5.78^ax^
	50	5.73^abx^	5.62^abxy^	5.53^aby^
	100	5.43^bx^	5.01^aby^	4.63^bz^
	200	4.73^cx^	4.23^by^	3.64^cz^

Shaking	Control	5.83^ax^	5.74^ax^	5.85^ax^
	50	3.49^dx^	ND	ND
	100	2.41^ex^	ND	ND
	200	1.29^fx^	ND	ND

^a–f^Means in the same column followed by different superscripts are significantly different at *P* < 0.05 (Duncan's multiple range test).

^x–z^Means in the same row followed by different superscripts are significantly different at *P* < 0.05 (Duncan's multiple range test).

**Table 4:** Coliform reduction of tilapia fillets disinfected by soaking or shaking treatments at different chlorine dioxide concentrations.

Wash method	Concentration (ppm)	Cleaning time (min)		
		5	15	25
		Coliforms log (CFU/g)		
Soaking	Control	5.23^ax^	5.33^ax^	5.21^ax^
	50	4.80^bx^	3.22^bz^	4.06^by^
	100	4.17^cx^	2.33^cy^	ND
	200	3.23^dx^	1.23^dy^	ND

Shaking	Control	5.23^ax^	5.29^ax^	5.25^ax^
	50	1.78^ex^	ND	ND
	100	1.07^fx^	ND	ND
	200	ND	ND	ND

^a–f^Means in the same column followed by different superscripts are significantly different at *P* < 0.05 (Duncan's multiple range test).

^x–z^Means in the same row followed by different superscripts are significantly different at *P* < 0.05 (Duncan's multiple range test).

**Table 5:** *Escherichia coli* reduction of tilapia fillets disinfected by soaking or shaking treatments at different chlorine dioxide concentrations.

Wash method	Concentration (ppm)	Cleaning time (min)		
		5	15	25
		*E. coli *log (CFU/g)		
Soaking	Control	5.23^ax^	5.33^ax^	5.22^ax^
	50	3.08^bx^	2.21^by^	1.1^cz^
	100	2.25^cx^	1.14^cy^	ND
	200	1.13^dx^	ND	ND

Shaking	Control	5.56^ax^	5.51^ax^	5.58^ax^
	50	1.1^dx^	ND	ND
	100	ND	ND	ND
	200	ND	ND	ND

^a–d^Means in the same column followed by different superscripts are significantly different at *P* < 0.05 (Duncan's multiple range test).

^x–z^Means in the same row followed by different superscripts are significantly different at *P* < 0.05 (Duncan's multiple range test).

**Table 6:** Total trihalomethane (THM) residuals by GC/MS.

Item	Total trihalomethane^a^ (ppb)
Methyl chloride	ND
Dichloromethane	ND
Chloroform	ND

^a^The instrument detection limit was 0.12 ppb.
